# The mental health status of ethnocultural minorities in Ontario and their mental health care

**DOI:** 10.1186/s12888-016-0759-z

**Published:** 2016-02-26

**Authors:** Sherry L. Grace, Yongyao Tan, Robert A. Cribbie, Han Nguyen, Paul Ritvo, Jane Irvine

**Affiliations:** School of Kinesiology and Health Science, York University, Toronto, ON Canada; Cardiac Rehabilitation and Prevention Program, Toronto Rehabilitation Institute, University Health Network, Toronto, ON Canada; Department of Psychology, York University, Toronto, ON Canada; Department of Medicine, University of Toronto, Toronto, ON Canada; Bethune College 368 York University, 4700 Keele Street, Toronto, ON Canada

**Keywords:** Mental health, Ethnic groups, Emigrants and immigrants, Ontario

## Abstract

**Background:**

Mental disorders are a leading cause of disability and early mortality. The objective of this study was to describe and compare psychosocial indicators and mental health service use among ethnoculturally-diverse Ontarians.

**Methods:**

This is a cross-sectional analysis of the Ontario Health Study pilot investigation. Residents were mailed an invitation to one of 3 assessment centres (urban, rural and northern sites) from March 2009 to July 2010. Participants had an interview with a nurse and completed a questionnaire on a touchscreen kiosk. The questionnaire included sociodemographic items, and scales assessing symptoms of depressive symptoms (CES-D) and anxiety (GAD-7), social support (Lubben Social Network Scale), stressful life events, and mental health service use.

**Results:**

Eight thousand two hundred thirty-five residents participated, among whom 6652 (82.4 %) self-reported their ethnocultural background as White, 225 (2.8 %) as South Asian, 222 (2.8 %) East Asian, 214 (2.7 %) Southeast Asian, 197 (2.4 %) Black, and 28 (0.3 %) as Aboriginal. Based on their sociodemographic characteristics, participants from these ethnocultural minority groups were matched to White participants. Black participants reported significantly greater stressful life events than White participants (*p* = .04), particularly death (*p* < .05), divorce (*p* = .002) and financial difficulties (*p* < .001). East Asian participants reported significantly less social support than their White counterparts (*p* < .001), and this was not confounded by measurement variance. Mental health service use was significantly lower in all ethnocultural minorities except Aboriginals, when compared to White participants (*p* = .001).

**Conclusions:**

There is a high burden of psychosocial distress in several preponderant ethnocultural minorities in Ontario; many of whom are not accessing available mental health services.

## Background

As the second leading cause of human disability and premature death, mental disorders are a significant public health concern [1]. Current estimates suggest one in five Canadians will experience a mental illness in their lifetime [2, 3]. In addition, mental health problems account for $51 billion in annual spending [2], making it one of the costliest healthcare burdens in Canada [4].

Anxiety disorders and depressive symptoms are two of the most prevalent mental disorders. Both anxiety and depressive symptoms can take a chronic debilitating course, with depressive symptoms particularly related to increased morbidity and mortality from medical conditions and decreased quality of life, among other consequences [5, 6]. Beyond mental disorders, social support and stressful life events have also been shown to affect an individual’s mental health and psychosocial well-being [7]. Individuals need not suffer with mental illness in Canada without treatment, as evidence-based treatments for the most common mental disorders [8], can be accessed within the provincial healthcare systems.

The term “ethnocultural minority” describes people of a “culturally and linguistically-diverse background”, where culture consists of a system of values, norms, and beliefs that shape an individual’s daily experiences and behavior [9]. In Canada, the most populous ethnocultural groups are South Asian (e.g., India, Pakistan, Bangladesh, and Sri Lanka; 4.8 %), Aboriginal (4.3 %), Chinese (4.0 %), and Black (2.9 %). As per the 2011 National Household Survey, 6,775,800 individuals were foreign-born, representing 20.6 % of the total population [10], and approximately 250,000 people immigrate to Canada each year [11]. Individuals from South Asia are the largest and fastest growing group.

The literature regarding mental health in Canada illustrates that despite the country’s diversity, there are very few comprehensive research studies regarding psychosocial well-being or access to mental health services among ethnocultural groups [12]. Ethnocultural minorities experience greater exposure to the negative effects of some social determinants of health, such as income insecurity and social isolation [12]. Immigrants and refugees are separated from their social supports, which makes them at the increased risk of mental health problems and illnesses [13]. Visible minorities often face racial discrimination. Finally, Aboriginal peoples in Canada are known to suffer poorer mental health, and an alarmingly high rate of suicide is observed [14].

Stakeholders have called for better research into these populations in Canada, arguing that more in-depth investigation may uncover the key processes that give rise to mental health risk [15]. Accordingly, the objective of this study was to describe and compare depressive and anxiety symptoms, stressful life events, perceived social support and mental health service use among ethnoculturally-diverse Ontarians.

## Methods

### Design and procedure

This cross-sectional study was based on data from the Ontario Health Study (OHS) pilot. The OHS aims to investigate how lifestyle, environment and family history factors increase the risk of common chronic diseases such as cancer, diabetes, and heart disease [16]. Data were collected at three assessment centres from March 2009 to July 2010. Potential participants were invited to attend an assessment centre to participate via mail. At the centres, participants completed a touchscreen questionnaire which included psychosocial assessments. Participants also had an interview with a nurse to provide personal and family medical history and prescription medications. A data access application for this study was approved by the OHS. Ethics approval was secured through the York University and University of Toronto Research Ethics Boards.

### Participants

Participants were recruited from the communities of Mississauga, Oakville, Brampton, Burlington, Owen Sound, and Sudbury, Ontario, Canada. These communities were selected to achieve representation of rural (Owen Sound), northern (Sudbury), and urban (all others) Ontarians in the sample. The urban centres are in the Greater Toronto Area, where a large proportion of Canadian immigrants settle [10]. Potential participants (*N* = 306,041) were identified from purchased mailing lists. Eligible participants had to be residents of Ontario, between the ages of 35–69 years, and sufficiently proficient in English or French. Participants provided written informed consent for participation in the study.

### Measures

Sociodemographic characteristics and mental health were assessed in the self-report survey. Participants were asked to report their ethnocultural background from the following options: Aboriginal (e.g., First Nations, Métis or Inuit), Arab (e.g., Egypt, Iraq, Jordan, Lebanon), Black (African or Caribbean descent), East Asians (i.e., China, Japan, Korea, Taiwan), Filipino, Jewish, Latin American/Hispanic, South Asian (e.g., India, Sri Lanka, Pakistan, Bangladesh), Southeast Asian (e.g., Malaysia, Indonesia, Vietnam), West Asian (e.g., Turkey, Iran, Afghanistan), White and other. Finally, participants were also asked to rate their health status, on a scale from 1 = poor, to 5 = excellent.

#### Psychosocial Well-Being

Depressive and anxiety symptoms, social support, and stressful life events were assessed. Depressive symptoms were measured using the Center for Epidemiological Studies Depressive symptoms Scale (CES-D) [17]. It is a 20-item self-report scale designed to measure depressive symptomatology in the general population. Scores range from 0 to 60, where higher scores indicate more symptoms, weighted by frequency of occurrence during the past week. The threshold for elevated depressive symptoms on the CES-D-20 is often interpreted as a score of 16 or greater. The scale has been validated in African American, Asian American, Asian, Chinese, French, Greek, Hispanic, Japanese, Serbian, and East Indian samples [18, 19]. Good reliability, excellent internal consistency (coefficient alpha >0.85 in community samples and 0.9 in psychiatric samples), and good test-retest correlation (*r* = .65) have been demonstrated. The CES-D also has high sensitivity and specificity [20].

Anxiety symptoms were measured using the Generalized Anxiety Disorder scale (GAD-7) [21]. It consists of seven items, each of which is scored 0 to 3, providing a total severity score ranging from 0 to 21. Cutpoints of 5, 10, and 15 represent mild, moderate, and severe levels of anxiety on the scale. Although originally developed to assess generalized anxiety disorder, the GAD-7 has also proved to have good sensitivity and specificity as a screener for panic, social anxiety, and post-traumatic stress disorder [22].

The Lubben Social Network Scale (LSNS-6) [23] is designed to gauge perceived social support received by family and friends. Scores range from 0 to 30, with higher scores indicating a greater level of social support and low risk for isolation. Scores less than 6 on the family or friend subscales suggest marginal ties, with overall scores less than 12 indicative of social isolation. The 6-item version has been demonstrated to be reliable (Cronbach’s α = 0.83), and to discriminate between socially-connected versus isolated participants [23].

The Total Life Events Scale measures stressful life events in the past year. The scale administered was a slightly modified version of the scale administered by the United Kingdom Biobank [24] and INTERHEART [25]. It is a 12-item questionnaire where respondents are asked whether they have experienced a specific event. Scores range from 0 to 12, with higher scores indicating a greater impact of stressful life events.

Finally, participants were asked to report whether they had visited a mental healthcare provider for “nerves, anxiety, tension or depressive symptoms” and to report which type of provider. This item was investigator-generated. Participants could check all the response alternatives that were applicable.

### Statistical analyses

IBM SPSS Statistics Software Version 21 was used for preliminary statistical analyses. Ethnocultural backgrounds for which there were more than 150 participants were selected for further analysis. This size was chosen so that upon propensity-matching, a sufficient sample would be available for robust comparison to White participants. However, Aboriginal participants were also selected for analysis regardless of size, given the known mental health disparities between Aboriginals and the general population [14]. A comparison of participant sociodemographic characteristics by ethnocultural background was performed.

In order to compare the White participants to the most common ethnocultural minority groups and Aboriginal participants, we matched the participants on important sociodemographic variables known to be related to mental health. Using the variables of sex, education, house ownership and income, participants were matched using the R package *MatchIt* [26]. Given the categorical nature of the demographic variables, exact matching was utilized.

To test for ethnocultural differences in psychosocial indicators, for the categorical outcome variables, Fisher’s exact test was used to compare frequencies across groups. This was undertaken for both the non-matched and matched samples, to enable a more fulsome understanding of the robustness of any associations observed. For continuous outcome variables the heteroscedastic Welch *t* test was used to compare the ethnocultural groups (non-matched and matched). For the variables depressive symptoms, anxiety, stressful life events and total mental health services use, the square root of the variables was used, since this brought the distribution of the variables towards normality.

In order to investigate the role that measurement invariance might play in psychosocial differences between the ethnocultural groups, if the matched groups differed on the well-validated scales, namely the CES-D, GAD-7 or Lubben scales, a multiple group structural equation model was used to determine if any of the loadings or factor covariances differed [27]. Item and factor intercepts/means were not assessed for invariance since those relate to the main hypotheses of the study. Model fit was assessed using the model chi-square statistic, root mean square error of approximation (RMSEA) and comparative fit index (CFI). For the RMSEA, values less than .06 are indicative of a good fitting model, and for the CFI values greater than .95 are indicative of a good fitting model (Hu & Bentler, 1999). These analyses were undertaken using R version 3.1.0 [28].

## Results

### Respondent characteristics

The sample consisted of 8235 participants (2.69 % response rate). Of the 259,159 urban Ontarians mailed, 5,228 (2.02 %) participated; of the 13,456 rural Ontarians mailed, 1,366 (10.2 %) participated; and of the 33,417 northern Ontarians mail, 1,641 (4.91 %) participated.

Table 1 describes self-reported ethnocultural background of participants. As shown, the most frequent ethnocultural backgrounds were White, South Asian, East Asian, Southeast Asian, and Black. This is somewhat consistent with the distribution of visible minorities in Ontario, namely South Asian (26 %), East Asian (22 %), Black (19 %), Filipino (7 %), Latin American (5 %), Arab (4 %), Southeast Asian (4 %), West Asian (3 %), and Korean (3 %), among others (http://www.fin.gov.on.ca/en/economy/demographics/census/cenhi6e.pdf). The proportion of Aboriginal respondents was 0.3 %, compared to 2.4 % in Ontario broadly [29].

**Table 1:** Self-reported ethnocultural background of OHS pilot study respondents

	*n*	%
White	6652	82.4
South Asian	225	2.8
East Asian	222	2.8
Southeast Asian	214	2.7
Black	197	2.4
Latin American/Hispanic	102	1.3
Filipino	66	0.8
Arab	43	0.5
Jewish	32	0.4
Aboriginal	28	0.3
West Asian	20	0.2
Multiple Ethnicities	193	2.2
Other	86	1.1

Participants’ sociodemographic characteristics are shown in Table 2. Overall, participants were a mean of 55.64 ± 8.89 years old (standard deviation). Also shown in Table 2 is a comparison of participants’ sociodemographic characteristics by ethnocultural background. Compared to White participants, there were significantly fewer female South Asian, East Asian and Southeast Asian participants, and significantly more female Aboriginal participants. With regard to highest educational attainment, East Asian and Southeast Asian participants reported significantly higher education than White participants. With regard to first language spoken, significantly more White participants reported English as their first language than South Asian, East Asian and Southeast Asian. However, Black participants were significantly more likely to report English as their first language than White participants. With regard to marital status, significantly more South Asian, East Asian and Southeast Asian participants reported being married or living with a partner than White participants. However, Black participants were significantly less likely than White participants to report being married or living with a partner. With regard to work status, South Asian, East Asian, Southeast Asian, and Black participants were significantly more likely to be working full-time than their White counterparts. With regard to household income, significantly more Black and Aboriginal participants reported earning less than $100,000 CAD annually than White participants. With regard to home ownership status, significantly fewer Black participants reported owning a home than White participants. With regard to health status, significantly fewer South Asian, East Asian, Southeast Asian and Black participants reported their health in general is Good or better than White participants.

**Table 2:** Characteristics of participants, by ethnocultural background

	n (%)						
	White 6652 (82.4)	South Asian 225 (2.8)	East Asian 222 (2.8)	Southeast Asian 214 (2.7)	Black 197 (2.4)	Aboriginal 28 (0.3)	Total *N* = 7538 (100)
Sex (Female)	3716 (55.9)	81 (36.0) (*p* < 0.001)	94 (42.3) (*p* < 0.001)	68 (31.8) (*p* < 0.001)	122 (61.9)	21 (75.0) (*p* < 0.001)	4102 (54.4)
Education (Greater than high school)	5182 (78.2)	186 (83.0)	199 (89.6) (*p* < 0.001)	193 (90.6) (*p* < 0.001)	155 (78.7)	18 (64.3)	5933 (79.0)
First Language (English)	5493 (82.6)	95 (42.4) (*p* < 0.001)	49 (22.3) (*p* < 0.001)	94 (43.9) (*p* < 0.001)	185 (94.4) (*p* < 0.001)	21 (75.0)	5937 (78.8)
Marital Status (Married or living with a partner)	5363 (80.7)	206 (92.0) (*p* < 0.001)	199 (89.6) (*p* < 0.001)	198 (92.5) (*p* < 0.001)	122 (62.2) (*p* < 0.001)	25 (89.3)	6113 (81.2)
Work Status (Full-time)	3069 (46.1)	150 (66.7) (*p* < 0.001)	136 (61.3) (*p* < 0.001)	141 (65.9) (*p* < 0.001)	124 (62.9) (*p* < 0.001)	14 (50.0)	3634 (48.2)
Household Income (<$100, 000 CAD)	3497 (52.6)	122 (54.2)	112 (50.5)	127 (59.3)	121 (61.4) (*p* = 0.008)	21 (75.0) (*p* = 0.008)	4000 (53.1)
Own Home (yes)	6260 (94.2)	207 (92.4)	213 (96.4)	201 (94.4)	175 (89.3) (*p* = 0.043)	24 (85.7)	7080 (94.1)
Perceived Health (Good to Excellent)	6332 (95.3)	195 (86.7) (*p* < 0.001)	198 (89.6) (*p* < 0.001)	191 (89.3) (*p* < 0.001)	175 (89.7) (*p* < 0.001)	25 (89.3)	7116 (94.5)

Note: *p* values show comparison between Non-White and White participants

Of the complete cases, 100 (96.2 %) of the 104 Black, 111 (98.2 %) of the 113 South Asian, 132 (98.5 %) of the 134 East Asian, 106 (95.5 %) of the 111 Southeast Asian, and 20 (95.2 %) of the 21Aboriginal participants had matches with the White participants. Of the 5120 White participants, 2083 (40.7 %) matched the characteristics of the included Black participants, 2115 (41.3 %) matched the characteristics of the included South Asian participants, 2486 (48.6 %) matched the characteristics of the included East Asian participants, 1898 (37.1 %) matched the characteristics of the included Southeast Asian participants, and 518 (10.1 %) matched the characteristics of the included Aboriginal participants.

### Psychosocial well-being

In the overall sample, 3471 (46.4 %) participants reported elevated depressive symptoms (i.e., CES-D scores greater than 16). With regard to anxiety, 1022 (13.6 %) participants reported mild, 324 (4.3 %) participants reported moderate, and 153 (2.0 %) participants reported severe symptoms. With regard to social ties, 1227 (15.4 %) participants would be considered marginally-tied to family, and 1476 (18.5 %) would be considered marginally-tied with friends. Overall, 1225 (15.5 %) scored as socially-isolated. On average, participants reported experiencing 1.6 ± 1.4 stressful life events in the past year, and 940 (12.3 %) reported they had experienced stressful life events other than those listed. Finally, 1749 (21.2 %) participants reported seeing any health care provider for mental health concerns, and where they did seek care it was most often received by a family medical doctor (*n* = 1027; 58.7 %), counsellor (*n* = 256, 14.6 %) or psychologist (*n* = 176; 10.1 %).

Ethnocultural comparisons across all participants and the matched samples can be found in Table 3 for depressive symptoms, anxiety, social support, stressful life events and mental health service usage. As shown, Black participants reported significantly more stressful life events and less mental health service use than White participants, in both the overall and matched samples. South Asian participants reported significantly greater anxiety and less mental health service use than White participants in both the overall and matched samples. East Asian participants reported significantly lower social support and mental health service use than White participants in both the overall and matched samples. Southeast Asian participants reported significantly less mental health service use than White participants in both the overall and matched samples. Finally, in the overall sample, Aboriginal participants reported significantly greater depressive symptoms, and a trend toward greater mental health service use than White participants. In the matched samples, there were trends towards greater depressive symptoms, anxiety and stressful life events among Aboriginal participants when compared to White participants, and also a trend toward lower social support.

**Table 3:** Comparison of psychosocial indicators between White and ethnocultural minority participants, in the overall and matched samples

Outcome Variable	All Data			Matched Data		
	White *n* = 5120	Black *n* = 104	*p*	White *n* = 2083	Black *n* = 100	*p*
Depressive symptoms	16.38 (6.26)	16.25 (6.60)	.668	16.51 (5.95)	16.11 (6.63)	.370
Anxiety	2.30 (3.46)	2.69 (4.16)	.829	2.19 (3.25)	2.80 (4.47)	.726
Life Events	1.53 (1.36)	1.88 (1.52)	.032	1.55 (1.37)	1.91 (1.53)	.041
Social Support	17.59 (5.40)	17.81 (5.92)	.708	17.79 (5.40)	18.01 (5.87)	.709
Mental Health Services Use	.63 (1.00)	.33 (.60)	<.001	.64 (1.00)	.33 (.60)	<.001
	White *n* = 5120	South Asian *n* = 113	*p*	White *n* = 2115	South Asian *n* = 111	*p*
Depressive symptoms	16.38 (6.26)	16.31 (6.25)	.870	16.06 (5.88)	16.22 (6.22)	.889
Anxiety	2.30 (3.46)	3.27 (4.78)	.048	2.19 (3.19)	3.27 (4.82)	.048
Life Events	1.53 (1.36)	1.65 (1.44)	.302	1.43 (1.29)	1.67 (1.44)	.088
Social Support	17.59 (5.40)	17.41 (6.11)	.756	17.55 (5.25)	17.45 (6.14)	.862
Mental Health Services Use	.63 (1.00)	.23 (.57)	<.001	.57 (.95)	.23 (.57)	<.001
	White *n* = 5120	East Asian *n* = 134	*p*	White *n* = 2486	East Asian *n* = 132	*p*
Depressive symptoms	16.38 (6.26)	16.37 (5.70)	.792	16.11 (5.82)	16.36 (5.74)	.533
Anxiety	2.30 (3.46)	2.11 (3.20)	.389	2.10 (3.07)	2.05 (3.15)	.525
Life Events	1.53 (1.36)	1.47 (1.37)	.608	1.43 (1.31)	1.44 (1.36)	.998
Social Support	17.59 (5.40)	14.72 (5.50)	<.001	17.44 (5.40)	14.73 (5.53)	<.001
Mental Health Services Use	.63 (1.00)	.20 (.49)	<.001	.58 (.97)	.20 (.49)	<.001
	White *n* = 5120	Southeast Asian *n* = 111	*p*	White *n* = 1898	Southeast Asian *n* = 106	*p*
Depressive symptoms	16.38 (6.26)	15.63 (7.36)	.117	16.06 (5.80)	15.48 (7.39)	.159
Anxiety	2.30 (3.46)	2.64 (4.16)	.829	2.11 (3.06)	2.57 (4.20)	.808
Life Events	1.53 (1.36)	1.42 (1.50)	.211	1.44 (1.33)	1.37 (1.46)	.347
Social Support	17.59 (5.40)	16.90 (5.93)	.229	17.21 (5.46)	16.82 (5.99)	.512
Mental Health Services Use	.63 (1.00)	.27 (.57)	<.001	.55 (.96)	.28 (.58)	.001
	White *n* = 5120	Aboriginal *n* = 21	*p*	White *n* = 518	Aboriginal *n* = 20	*p*
Depressive symptoms	16.38 (6.26)	22.00 (10.77)	.033	17.00 (6.30)	22.40 (10.89)	.055
Anxiety	2.30 (3.46)	4.19 (5.11)	.187	2.05 (3.13)	4.40 (5.14)	.092
Life Events	1.53 (1.36)	2.10 (1.37)	.116	1.64 (1.47)	2.20 (1.32)	.067
Social Support	17.59 (5.40)	15.76 (4.90)	.104	18.01 (5.38)	15.65 (5.01)	.052
Mental Health Services Use	.63 (1.00)	.95 (1.07)	.072	.75 (1.02)	.90 (1.07)	.367

Since the matched White and South Asian participants differed significantly on the GAD-7, a test of measurement invariance was conducted using a multiple group structural equation model. More specifically, a multiple group confirmatory factor analysis was conducted with a single anxiety factor. Error covariances were included between chronic worrying and uncontrolled worrying, feeling afraid and uncontrolled worrying, and difficulty relaxing and restlessness. It was found that the factor loadings varied across the groups. Specifically, the factor loadings of the South Asian participants were significantly higher than those of the White participants on chronic worrying, difficulty relaxing, feeling afraid, feeling restless and uncontrolled worrying. There were no differences between the groups on being easily annoyed or nervous. All standardized factor loadings for both groups were greater than .5. The final model fit the data very well (*χ*^2^ [24 df] = 177.01, *p* < .001, RMSEA = .035, CFI = .992). Therefore the greater anxiety reported by the South Asian participants should not be over-interpreted given the stronger relationships between many of the items within the South Asian participants (relative to the White participants).

Since the matched White and East Asian participants differed significantly on the social support scale, a test of measurement invariance was again conducted using a multiple group structural equation model. More specifically, a multiple group confirmatory factor analysis was conducted with separate factors for support from friends and family. Error covariances were included between the associated relative and friend items “number seen or heard from” and “number can talk to about private matters”. All factor loadings and the covariance between friend and family support were invariant across groups. Further, all standardized loadings for both groups were greater than .65. The final model had a good fit to the data (*χ*^2^ [12 df] = 230.05, *p* < .001, RMSEA = .043, CFI = .986).

Ethnocultural comparisons in specific stressful life events can be found in Table 4. In the matched samples, Black participants were significantly more likely to report experiencing the death of a partner, death or a relative or friend, divorce or separation, and financial difficulties in the last year than White participants. South Asians were significantly more likely to report business failure than White participants. Aboriginal participants were significantly more likely to report retirement than White participants in the matched sample, with a trend toward more death among family and friends in the matched sample, and family conflict in the overall sample.

**Table 4:** Comparison of specific stressful life events, by ethnocultural background, in overall and matched samples

Stressful Life Event	All Data			Matched Data		
	White *n* = 5120	Black *n* = 104	*p*	White *n* = 2083	Black *n* = 100	*p*
Illness/Injury	539 (10.5 %)	6 (5.8 %)	.143	228 (10.9 %)	6 (6.0 %)	.137
Illness/Injury to rel/frnd	2258 (44.1 %)	35 (33.7 %)	.036	936 (44.9 %)	35 (35.0 %)	.062
Death of a partner	46 (.9 %)	3 (2.9 %)	.073	15 (.7 %)	3 (3.0 %)	.046
Death of a rel/frnd	1505 (29.4 %)	44 (42.3 %)	.006	619 (29.7 %)	43 (43.0 %)	.007
Divorce/Sep	146 (28.5 %)	10 (9.6 %)	.001	65 (3.1 %)	10 (10 %)	.002
Finance diff	968 (18.9 %)	40 (38.5 %)	<.001	376 (18.1 %)	38 (38 %)	<.001
Job Loss	359 (7.0 %)	11 (10.6 %)	.173	135 (6.5 %)	10 (10.0 %)	.212
Retirement	473 (9.2 %)	9 (8.7 %)	.999	175 (8.4 %)	8 (8.0 %)	.999
Crop Loss	30 (.6 %)	1 (1.0 %)	.465	15 (.7 %)	1 (1.0 %)	.529
Business Failure	72 (1.4 %)	1 (1.0 %)	.999	37 (1.8 %)	1 (1.0 %)	.999
Family Conflict	869 (17.0 %)	21 (20.2 %)	.359	369 (17.7 %)	21 (21.0 %)	.422
	White *n* = 5120	South Asian *n* = 113	*p*	White *n* = 2115	South Asian *n* = 111	*p*
Illness/Injury	539 (10.5 %)	12 (10.6 %)	.999	212 (10.0 %)	12 (10.8 %)	.747
Illness/Injury to rel/frnd	2258 (44.1 %)	55 (48.7 %)	.340	890 (42.1 %)	55 (49.5 %)	.139
Death of a partner	46 (.9 %)	1 (.9 %)	.999	13 (.6 %)	1 (.9 %)	.512
Death of a rel/frnd	1505 (29.4 %)	36 (31.9 %)	.602	590 (27.9 %)	35 (31.5)	.448
Divorce/Sep	146 (28.5 %)	2 (1.8 %)	.772	49 (2.3 %)	2 (1.8 %)	.999
Finance diff	968 (18.9 %)	24 (21.2 %)	.544	356 (16.8 %)	23 (20.7 %)	.300
Job Loss	359 (7.0 %)	11 (9.7 %)	.263	171 (8.1 %)	11 (9.9 %)	.476
Retirement	473 (9.2 %)	8 (7.1 %)	.513	203 (9.6 %)	8 (7.2 %)	.506
Crop Loss	30 (.6 %)	1 (.9 %)	.493	15 (.7 %)	1 (.9 %)	.560
Business Failure	72 (1.4 %)	10 (8.8 %)	<.001	24 (1.1 %)	10 (9.0 %)	<.001
Family Conflict	869 (17.0 %)	14 (12.4 %)	.252	319 (15.1 %)	14 (12.6 %)	.585
	White *n* = 5120	East Asian *n* = 134	*p*	White *n* = 2486	East Asian *n* = 132	*p*
Illness/Injury	539 (10.5 %)	11 (8.2 %)	.475	245 (9.9 %)	11 (8.3 %)	.654
Illness/Injury to rel/frnd	2258 (44.1 %)	57 (42.5 %)	.792	1056 (42.5 %)	55 (41.7 %)	.928
Death of a partner	46 (.9 %)	2 (1.5 %)	.348	17 (.7 %)	2 (1.5 %)	.248
Death of a rel/frnd	1505 (29.4 %)	47 (35.1 %)	.179	707 (28.4 %)	45 (34.1 %)	.168
Divorce/Sep	146 (28.5 %)	2 (2.9 %)	.591	56 (2.3 %)	2 (1.5 %)	.999
Finance diff	968 (18.9 %)	20 (14.9 %)	.265	412 (16.6 %)	18 (13.6 %)	.469
Job Loss	359 (7.0 %)	10 (7.5 %)	.863	164 (6.6 %)	10 (7.6 %)	.593
Retirement	473 (9.2 %)	11 (8.2 %)	.879	220 (8.8 %)	11 (8.3 %)	.999
Crop Loss	30 (.6 %)	1 (.7 %)	.552	15 (.6 %)	1 (.8 %)	.564
Business Failure	72 (1.4 %)	5 (3.7 %)	.046	42 (1.7 %)	5 (3.8 %)	.085
Family Conflict	869 (17.0 %)	17 (12.7 %)	.242	378 (15.2 %)	16 (12.1 %)	.383
	White *n* = 5120	Southeast Asian *n* = 111	*p*	White *n* = 1898	Southeast Asian *n* = 106	*p*
Illness/Injury	539 (10.5 %)	12 (10.8 %)	.876	184 (.097)	12 (11.3 %)	.613
Illness/Injury to rel/frnd	2258 (44.1 %)	35 (31.5 %)	.009	808 (42.6 %)	33 (31.1 %)	.020
Death of a partner	46 (.9 %)	0 (0 %)	.627	9 (.5 %)	0 (0 %)	.999
Death of a rel/frnd	1505 (29.4 %)	31 (27.9 %)	.833	533 (28.1 %)	28 (26.4 %)	.824
Divorce/Sep	146 (28.5 %)	2 (1.8 %)	.771	50 (2.6 %)	2 (1.9 %)	.999
Finance diff	968 (18.9 %)	30 (27.0 %)	.037	315 (16.6 %)	27 (25.5 %)	.024
Job Loss	359 (7.0 %)	14 (12.6 %)	.037	137 (7.2 %)	12 (11.3 %)	.126
Retirement	473 (9.2 %)	5 (4.5 %)	.095	180 (9.5 %)	5 (4.7 %)	.119
Crop Loss	30 (.6 %)	1 (.9 %)	.487	16 (.8 %)	0 (0 %)	.999
Business Failure	72 (1.4 %)	2 (1.8 %)	.671	33 (1.7 %)	2 (1.9 %)	.708
Family Conflict	869 (17.0 %)	11 (9.9 %)	.053	289 (15.2 %)	10 (9.4 %)	.123
	White *n* = 5120	Aboriginal *n* = 21	*p*	White *n* = 518	Aboriginal *n* = 20	*p*
Illness/Injury	539 (10.5 %)	3 (14.3 %)	.480	60 (11.6 %)	3 (15 %)	.719
Illness/Injury to rel/frnd	2258 (44.1 %)	12 (57.1 %)	.273	246 (47.5 %)	12 (60.0 %)	.362
Death of a partner	46 (.9 %)	0 (0 %)	.999	6 (1.2 %)	0 (0 %)	.999
Death of a rel/frnd	1505 (29.4 %)	10 (47.6 %)	.090	163 (31.5 %)	10 (50.0 %)	.091
Divorce/Sep	146 (28.5 %)	0 (0 %)	.999	16 (3.1 %)	0 (0 %)	.999
Finance diff	968 (18.9 %)	4 (19.0 %)	.999	102 (19.7 %)	4 (20.0 %)	.999
Job Loss	359 (7.0 %)	0 (0 %)	.396	36 (6.9 %)	0 (0 %)	.387
Retirement	473 (9.2 %)	4 (19.0 %)	.124	35 (6.8 %)	4 (20.0 %)	.049
Crop Loss	30 (.6 %)	0 (0 %)	.999	4 (.8 %)	0 (0 %)	.999
Business Failure	72 (1.4 %)	0 (0 %)	.999	10 (1.9 %)	0 (0 %)	.999
Family Conflict	869 (17.0 %)	7 (33.3 %)	.072	103 (19.9 %)	7 (35.0 %)	.151

rel: a close relative; frnd: friend; Sep: separation; diff: difficulties

Ethnocultural comparisons in types of mental healthcare provider accessed can be found in Table 5. This was considered in all the most common ethnocultural groups as significant differences were observed in use overall, and also among Aboriginal participants where a trend was observed. Black participants were significantly less likely to see a psychiatrist than their White counterparts. South Asian and East Asian participants were significantly less likely to see a psychiatrist or a family doctor than their White counterparts. Southeast Asian participants were significantly less likely to see a counsellor than their White counterparts. Finally, Aboriginal participants were significantly more likely to see a psychologist than White participants.

**Table 5:** Comparison of specific mental healthcare provider accessed, by ethnocultural background, in overall and matched samples

Mental Health Care Provider Accessed	All Data			Matched Data		
	White *n* = 5120	Black *n* = 104	*p*	White *n* = 2083	Black *n* = 100	*p*
Psychiatrist	535 (10.4 %)	3 (2.9 %)	.008	224 (10.8 %)	3 (3.0 %)	.011
Psychologist	409 (8.0 %)	3 (2.9 %)	.063	158 (7.6 %)	3 (3.0 %)	.114
Psychotherapist	169 (3.3 %)	2 (1.9 %)	.777	72 (3.5 %)	2 (2.0 %)	.774
Social Worker	200 (3.9 %)	1 (1.0 %)	.190	85 (4.1 %)	1 (1.0 %)	.182
Counsellor	553 (10.8 %)	6 (5.8 %)	.110	235 (11.2 %)	6 (6.0 %)	.139
Nurse	34 (0.7 %)	0 (0 %)	.999	14 (.7 %)	0 (0 %)	.999
Family Doctor	1324 (25.9 %)	19 (18.3 %)	.089	540 (25.9 %)	18 (18.0 %)	.079
	White *n* = 5120	South Asian *n* = 113	*p*	White *n* = 2115	South Asian *n* = 111	*p*
Psychiatrist	535 (10.4 %)	3 (2.7 %)	.004	203 (9.6 %)	3 (2.7 %)	.011
Psychologist	409 (8.0 %)	5 (4.4 %)	.215	160 (7.6 %)	4 (3.6 %)	.137
Psychotherapist	169 (3.3 %)	1 (.9 %)	.273	51 (2.4 %)	1 (.9 %)	.516
Social Worker	200 (3.9 %)	1 (.9 %)	.132	74 (3.5 %)	1 (.9 %)	.179
Counsellor	553 (10.8 %)	5 (4.4 %)	.029	207 (9.8 %)	5 (4.5 %)	.068
Nurse	34 (0.7 %)	0 (0 %)	.999	10 (.5 %)	0 (0 %)	.999
Family Doctor	1324 (25.9 %)	11 (9.7 %)	<.001	510 (24.1 %)	11 (9.9 %)	<.001
	White *n* = 5120	East Asian *n* = 134	*p*	White *n* = 2486	East Asian *n* = 132	*p*
Psychiatrist	535 (10.4 %)	5 (3.7 %)	.009	256 (10.3 %)	4 (3.0 %)	.004
Psychologist	409 (8.0 %)	8 (6.0 %)	.516	185 (7.4 %)	8 (6.1 %)	.732
Psychotherapist	169 (3.3 %)	3 (2.2 %)	.803	69 (2.8 %)	3 (2.3 %)	.999
Social Worker	200 (3.9 %)	2 (1.5 %)	.248	89 (3.6 %)	2 (1.5 %)	.324
Counsellor	553 (10.8 %)	1 (.7 %)	<.001	248 (10.0 %)	1 (.8 %)	<.001
Nurse	34 (0.7 %)	0 (0 %)	.999	14 (.6 %)	0 (0 %)	.999
Family Doctor	1324 (25.9 %)	8 (6.0 %)	<.001	569 (22.9 %)	8 (6.1 %)	<.001
	White *n* = 5120	Southeast Asian *n* = 111	*p*	White *n* = 1898	Southeast Asian *n* = 106	*p*
Psychiatrist	535 (10.4 %)	4 (3.6 %)	.017	171 (9.0 %)	4 (3.8 %)	.074
Psychologist	409 (8.0 %)	3 (2.7 %)	.047	142 (7.5 %)	3 (2.8 %)	.081
Psychotherapist	169 (3.3 %)	0 (0 %)	.052	46 (2.4 %)	0 (0 %)	.172
Social Worker	200 (3.9 %)	1 (.9 %)	.131	65 (3.4 %)	.9 %)	.258
Counsellor	553 (10.8 %)	2 (1.8 %)	.001	182 (9.6 %)	2 (1.9 %)	.005
Nurse	34 (0.7 %)	0 (0 %)	.999	10 (.5 %)	0 (0 %)	.999
Family Doctor	1324 (25.9 %)	20 (18.0 %)	.062	425 (22.4 %)	20 (18.9 %)	.471
	White *n* = 5120	Aboriginal *n* = 21	*p*	White *n* = 518	Aboriginal *n* = 20	*p*
Psychiatrist	535 (10.4 %)	3 (14.3 %)	.477	62 (12.0 %)	2 (10.0 %)	.999
Psychologist	409 (8.0 %)	5 (23.8 %)	.023	42 (8.1 %)	5 (25.0 %)	.023
Psychotherapist	169 (3.3 %)	0 (0 %)	.999	22 (4.2 %)	0 (0 %)	.999
Social Worker	200 (3.9 %)	0 (0 %)	.999	19 (3.7 %)	0 (0 %)	.999
Counsellor	553 (10.8 %)	3 (14.3 %)	.490	75 (14.5 %)	3 (15.0 %)	.999
Nurse	34 (0.7 %)	0 (0 %)	.999	516 (0.4 %)	0 (0 %)	.999
Family Doctor	1324 (25.9 %)	9 (42.9 %)	.084	169 (32.6 %)	8 (40.0 %)	.477

## Discussion

The OHS serves as a rich source of information on the mental health status of ethnocultural minorities in Ontario. The findings likely reflect the experience of first and second generation immigrants to Ontario (e.g., South and Southeast Asian), but also reflect the experiences of long-established residents (e.g., Black, Aboriginal) who face a documented social disadvantage. Results showed that the most common ethnocultural minorities suffer a greater burden of psychosocial distress than their White counterparts, and, with the exception of Aboriginal Ontarians, were less likely to access care to treat this distress.

The literature regarding Canadian mental health more broadly illustrates that despite national diversity, few comprehensive studies have focused on mental health risk in relation to ethnicity. In addition, the overwhelming majority of such studies have focused specifically on depressive symptoms [12]. Previous studies have reported complex and variable associations when adults of specific national origins have been compared [30–37]. For example, higher rates of depressive symptoms have been reported in specific ethnic and sex-defined populations, like Chinese, Vietnamese, Portuguese, and Latin American women, compared to national figures [38]. In a study of Canadians of Ethiopian origin, Fenta et al. found rates that were lower or comparable to the national figures [39]. A number of studies have reported a lower prevalence of depressive symptoms in immigrants than those who are Canadian-born [12], in accordance with the so-called “healthy immigrant effect”. Taken together, these data suggest that while ethnicity itself may not be predictive of depressive and anxiety rates, psychosocial factors associated with acculturation and social disadvantage may contribute to the increased mental health risk seen in ethnic minority populations.

In this study, ethnocultural minorities reported lower access to mental health care providers. Given that mental health risk has been illustrated to be higher in these groups, minority Ontarians, even under universal health care, are likely suffering psychological distress without accessing effective treatments. Previous research has similarly shown that ethnic minorities make less use of services than do the White majority, although there is limited understanding as to why this is the case [40]. Factors such as inability to effectively communicate and navigate within Canada’s health care system may play a role. Insufficient access could also be linked to tendencies in some ethnocultural groups to seek healthcare that addresses physical illness but not for mental health, screening or preventive care services. There may also be ethnocultural differences associated with the propensity to seek support from religious sources, in contrast to secular sources, for mental health problems [41].

Caution is warranted in interpreting the findings. First, the generalizability is limited by the low response rate. The generalizability of the sample to the population of Ontario more broadly remains unknown. Moreover, given that the surveys were administered in English, generalizability is limited to immigrants with English-language proficiency. Second, no structured diagnostic interviews were performed, and while psychometrically-validated measures of depressive symptoms and anxiety in particular were administered, it is unknown how many participants would be diagnosed with a true mental disorder. Third, the lack of data on place of birth and generation status, immigration/refugee status, years in Canada, and English-language proficiency in the OHS dataset limits the potential to interpret these findings. Finally, multiple comparisons were performed, which may have inflated type I error.

## Conclusion

Mental health disparities are observed in Ontarians, with the most common or prevalent ethnocultural minorities suffering a higher level of psychosocial distress than their White counterparts. Furthermore, with the exception of Aboriginal Ontarians, these ethnocultural minorities are also less likely to access treatments that address distress. In the context of few Canadian studies on mental health risk in relation to ethnicity, our study indicates that the social determinants of health may be important factors to consider in future.

## Abbreviations

CES-D: Center for Epidemiological Studies Depressive Symptoms Scale
GAD-7: Generalized Anxiety Disorder Scale
OHS: Ontario Health Study
